# Different COVID-19 outcomes among systemic rheumatic diseases: a nation-wide cohort study

**DOI:** 10.1093/rheumatology/keac422

**Published:** 2022-08-03

**Authors:** Vasiliki-Kalliopi Bournia, George E Fragoulis, Panagiota Mitrou, Konstantinos Mathioudakis, Anastasios Tsolakidis, George Konstantonis, Ioulia Tseti, Georgia Vourli, Maria G Tektonidou, Dimitrios Paraskevis, Petros P Sfikakis

**Affiliations:** Joint Academic Rheumatology Program, National and Kapodistrian University of Athens, Medical School; Joint Academic Rheumatology Program, National and Kapodistrian University of Athens, Medical School; Hellenic Ministry of Health; IDIKA SA-e-Government Center for Social Security Services, Athens; IDIKA SA-e-Government Center for Social Security Services, Athens; Joint Academic Rheumatology Program, National and Kapodistrian University of Athens, Medical School; Uni-Pharma S.A., Kifissia; Department of Hygiene Epidemiology and Medical Statistics, Medical School, National and Kapodistrian University of Athens, Athens, Greece; Joint Academic Rheumatology Program, National and Kapodistrian University of Athens, Medical School; Department of Hygiene Epidemiology and Medical Statistics, Medical School, National and Kapodistrian University of Athens, Athens, Greece; Joint Academic Rheumatology Program, National and Kapodistrian University of Athens, Medical School

**Keywords:** COVID-19, RA, AS, PsA, SLE, SSc

## Abstract

**Objectives:**

To investigate coronavirus disease 2019 (COVID-19)-associated risk of hospitalization and death in RA, AS, PsA, SLE and SSc in comparison with the general population during the first year of the pandemic, and compare their overall mortality with 2019.

**Methods:**

Interlinking nationwide electronic registries, we recorded confirmed COVID-19-associated infections, hospitalizations and deaths, and all-cause deaths between 1 March 2020 and 28 February 2021 in all adults with RA, AS, PsA, SLE and SSc under treatment (*n* = 74 970, median age 67.5, 51.2, 58.1, 56.2 and 62.2 years, respectively) and in random comparators from the general population matched (1:5) on age, sex and region of domicile. Deaths from all causes during 2019 were also recorded.

**Results:**

Compared with the general population, incidence rates (IR) for COVID-19-associated hospitalization were higher in RA [IR ratio (IRR) 1.71(1.50–1.95)], SLE [2.0 (1.4–2.7)] and SSc [2.28 (1.29–3.90)], while COVID-19-associated death rates were higher in RA [1.91 (1.46–2.49)]. When focusing only on severe acute respiratory syndrome coronavirus 2–infected subjects, after adjusting for age and gender, the odds ratio for COVID-19 associated death was higher in RA [1.47 (1.11–1.94)] and SSc [2.92 (1.07–7.99)] compared with the general population. The all-cause mortality rate compared with the general population increased in RA during the first year of the pandemic (IRR 0.71) with reference to 2019 (0.59), and decreased in SSc (IRR 1.94 *vs* 4.36).

**Conclusion:**

COVID-19 may have a more severe impact in patients with systemic rheumatic disease than in the general population. COVID-19-related mortality is increased in subgroups of patients with specific rheumatic diseases, underscoring the need for priority vaccination and access to targeted treatments.

Rheumatology key messages

- RA, SLE and SSc patients have higher COVID-19-associated hospitalization risk compared with the general population.
- RA patients have higher risk of COVID-19-associated death compared with the general population.
- All-cause mortality during pandemic-associated lockdown increased *vs* 2019 in RA and decreased in SSc.

## Introduction

Over the last 2 years, coronavirus disease 2019 (COVID-19) has been associated with increased morbidity and mortality in the general population [1]. However, evidence regarding systemic rheumatic diseases which affect almost 2% of the population [2] is not conclusive. In the systematic literature review performed to inform the respective EULAR recommendations, no signal for increased mortality in systemic rheumatic diseases compared with the general population was detected [3]. Similar results were presented in a recent meta-analysis examining data from 26 observational studies [4]. On the other hand, a large meta-analysis of 71 studies showed that patients with systemic rheumatic disease displayed increased odds for mortality [5]. The same uncertainty also applies to the question of whether systemic rheumatic disease patients are more susceptible to contracting COVID-19, with systematic literature reviews and meta-analyses again having discordant results [3–5]. To make matters more complicated, the known increased rates of concomitant comorbidities, including arterial hypertension, cardiovascular disease, diabetes and malignancy [6–12] may have a major additional impact. In any case, whether a propensity of systemic rheumatic disease patients towards more severe COVID-19-associated outcomes, as found in some studies, is true for some or for all diseases remains unclear.

During the first year of the pandemic, Greece implemented a strict lockdown policy limiting COVID-19-associated deaths to 6504. In this setting, by interlinking data from national electronic registries between March 2020, when the COVID-19 pandemic started in our country, and February 2021, when vaccination became available to patients with systemic rheumatic diseases, we aimed to investigate the COVID-19-associated risk of hospitalization and death in patients with RA, AS, PsA, SLE and SSc in comparison with the general population. Furthermore, we sought to assess the risk of COVID-19-associated hospitalization and death following infection, focusing only on severe acute respiratory syndrome coronavirus 2 (SARS-CoV-2)-infected subjects from our cohort, with or without underlying systemic rheumatic disease. Finally, we aimed to compare overall mortality of RA, AS, PsA, SLE and SSc during the first year of the pandemic with their overall mortality in the pre-COVID-19 year.

## Methods

### Setting

The first confirmed case of COVID-19 in Greece was identified on 26 February 2020. Until 28 February 2021 there had been 191 100 confirmed cases and 6504 COVID-19-associated deaths reported in the country. During the first year of the pandemic several measures were implemented to contain transmission of the virus, including a strict lockdown from 23 March 2020 until 14 May 2020; schools and tourist enterprises did not reopen until 1 June 2020 and 1 July 2020, respectively. A second pandemic wave in November 2020 led to a new lockdown from 7 November 2020, with measures remaining in place until after March 2021 in some regions, including Athens, the country’s capital. On the other hand, priority vaccination against COVID-19 in Greece became available for patients with systemic rheumatic disease in mid-March 2021.

### Data sources

In Greece, an electronic database for social security services (IDIKA) has operated since 2011, currently covering 99% of the country’s population of about 11 000 000 people. Information about prescribed medications, medical diagnoses [based on the specific International Classification of Diseases (ICD-10)], age, gender and region of domicile derived from this database can be linked to the nationwide death records as well as to the national COVID-19 digital registry, which includes data on hospitalizations and deaths of all confirmed COVID-19 cases in the country.

### Study population

In this nationwide, population-based cohort study we identified all adult patients with RA, AS, PsA, SLE and SSc alive on 1 March 2020. For this purpose, we used our published data [13] derived from the electronic prescription database for social security services (IDIKA). This data included all adult (aged ≥18 years old) patients who had filled at least one prescription for CS, conventional synthetic DMARDs, immunosuppressants, biologic DMARDs, targeted synthetic DMARDs, advanced vasodilatory medications or antifibrotic agents between 1 January 2015 and 31 December 2019 with a diagnosis of either RA, AS, PsA, SLE or SSc, based on prespecified for each disease ICD-10 codes. A detailed list of relevant ICD-10 (supplementary Table S1, available at *Rheumatology* online) and Anatomical Therapeutic Chemical-5 codes (supplementary Table S2, available at *Rheumatology* online) for diagnoses and medications of interest, respectively, is shown in the supplementary material available at *Rheumatology* online. Each of the 74 970 patients identified in total was matched to five random referents from the general population for gender and age, as well as for region of domicile.

For all subjects in our cohort, we retrieved data on age, gender and death from all causes and by crosslinking with the national COVID-19 registry, data on SARS-CoV-2 infection confirmed by reverse-transcriptase PCR or antigen-detecting rapid diagnostic testing, as well as COVID-19-associated hospitalization and death, during the study period, i.e. between 1 March 2020 and 28 February 2021.

To compare incidence rates of death from all causes during the first year of the pandemic in our cohort with the respective incidence rates from the pre COVID-19 era, we identified all adult patients with RA, AS, PsA, SLE and SSc alive on 1 January 2019 and again matched each patient to five random referents from the general population for gender, age and region of domicile, using the same methodology as described above. This comparison cohort included all patients aged 18 years or older, who had filled at least one prescription for the above-mentioned medications of interest with a diagnosis of RA, AS, PsA, SLE or SSc, as specified above, between 1 January 2015 and 31 December 2018. For these patients we recorded death from all causes between 1 January 2019 and 31 December 2019.

### Ethics approval and consent to participate

The Data Protection Office of the Greek Ministry of Health (17 Aristotelous str., 10187, Athens, Greece, email: dpo@moh.gov.g/gdpr@moh.gov.g), gave ethical approval for this work, granting permission for the use of anonymized data deposited in the social security services (IDIKA) database and the national COVID-19 digital registry, according to the European legislation for General Data Protection Regulation (27 April 2016) and the Greek national laws (4600/2019, 4624/2019, 3892/10, 3418/2005).

### Statistical analysis

Continuous variables were presented as median (Q1–Q3) and categorical variables were reported as numbers and percentages. Incidence rates (IR) per 1000 person-years and patient-to-referent incidence rate ratios (IRR) were estimated for (i) SARS-CoV-2 infection, (ii) COVID-19-associated hospitalization and (iii) COVID-19-related death for RA, AS, PsA, SLE and SSc patients and their matched referents from the general population. Binary logistic regression analysis was further conducted for each disease group and its referents, to estimate odd ratios (OR) for COVID-19 related hospitalization and death, in SARS-CoV-2-infected subjects in our cohort, using subjects free from underlying systemic rheumatic disease as the reference category. All logistic regression models were adjusted for age, gender and underlying disease. Incidence rates of death from all causes and patient-to-referent IRR were estimated for RA, AS, PsA, SLE and SSc patients and for their matched population referents during two different time periods, the first extending from 1 January 2019 to 31 December 2019 and the second from 1 March 2020 to 28 February 2021. In all analyses age was treated as a continuous variable, increasing by year. There were no missing data on covariates we selected to record. The level of statistical significance was set at a *P*-value of ≤0.05. Statistical analysis was performed using the Stata statistical software package (StataCorp. 2013; Stata Statistical Software: Release 13; StataCorp LP, College Station, TX, USA).

## Results

### Cohort demographics

Our search of the IDIKA database, using the above-described criteria, retrieved 40 014 RA patients (79% female), 9566 AS patients (43% female), 13 405 PsA patients (55% female), 9960 SLE patients (90% female) and 2025 SSc patients (88% female). RA patients were older compared with other systemic rheumatic disease patients, with a median (Q1–Q3) age of 67.5 (57.4–76.0) years, followed by SSc [62.2 (51.7–71.0) years], PsA [58.1 (48.6–68.0) years], SLE [56.2 (45.2–67.2) years] and AS [51.2 (41.9–60.4) years] patients. Total follow-up was 74 765 person-years for systemic rheumatic disease patients and 372 019 person-years for their matched comparators. Table 1 shows demographics of systemic rheumatic disease patients and their matched referents from the general population. SARS-CoV-2 infections, COVID-19-related hospitalizations and deaths occurring in our cohort between 1 March 2020 and 28 February 2021 are also shown in Table 1.

**Table 1:** Demographics and number of COVID-19-associated infections, hospitalizations, and deaths recorded among patients and population referents

	RA		AS		PsA		SLE		SSc	
	Patients	Population referents	Patients	Population referents	Patients	Population referents	Patients	Population referents	Patients	Population referents
	*N* = 40 014	*N* = 200 070	*N* = 9566	*N* = 47 830	*N* = 13 405	*N* = 67 025	*N* = 9960	*N* = 49 800	*N* = 2025	*N* = 10 125
Female gender, *N* (%)	31 782 (79.4)	158 910 (79.4)	41 287 (43.1)	20 635 (43.1)	7402 (55.2)	37 010 (55.2)	8948 (89.8)	44 740 (89.8)	1786 (88.2)	8930 (88.2)
Median age (Q1–Q3) at study entry, years	67.5 (57.4–76.0)		51.2 (41.9–60.4)		58.1 (48.6–68.0)		56.2 (45.2–67.2)		62.2 (51.7–71.0)	
SARS-CoV-2 infection, *N* (%)	767 (1.92)	2860 (1.43)	221 (2.31)	860 (1.80)	285 (2.13)	1156 (1.72)	197 (1.98)	812 (1.63)	49 (2.42)	161 (1.59)
COVID-19 hospitalization, *N* (%)	315 (0.79)	914 (0.46)	42 (0.44)	154 (0.32)	65 (0.48)	260 (0.39)	59 (0.59)	151 (0.30)	21 (1.04)	46 (0.45)
COVID-19 death, *N* (%)	79 (0.20)	205 (0.10)	3 (0.03)	21 (0.04)	10 (0.07)	41 (0.06)	9 (0.09)	25 (0.05)	5 (0.25)	9 (0.09)

Demographics and number of SARS-CoV-2 infections, number of COVID-19-associated hospitalizations and deaths recorded between 1 March 2020 and 28 February 2021 among patients with RA, AS, PsA, SLE and SSc and their matched population referents. COVID-19: coronavirus disease 2019; SARS-CoV-2: severe acute respiratory syndrome coronavirus 2.

### Comparison of patients with RA, AS, PsA, SLE and SSc with matched population referents

Table 2 shows IR per 1000 patient-years for COVID-19-associated infection, hospitalization and death among patients with RA, AS, PsA, SLE and SSc, and their matched population comparators. Rates of infection were higher among patients with systemic rheumatic diseases compared with their matched referents [IRR 1.33 (1.23–1.44) for RA, IRR 1.28 (1.10–1.49) for AS, IRR 1.23 (1.07–1.40) for PsA, IRR 1.21 (1.03–1.42) for SLE and IRR 1.52 (1.08–2.11) for SSc, respectively]. Regarding rates of hospitalization, these were higher among patients with RA [IRR 1.71 (1.50–1.95)], SLE [IRR 2.0 (1.4–2.7)] and SSc [IRR 2.28 (1.29–3.90)] compared with their matched population referents. Finally, mortality rates were only found to be higher among RA patients compared with their matched comparators from the general population [IRR 1.91 (1.46–2.49)].

**Table 2:** Patient to matched population referent incidence rate ratios of COVID-19-associated infections, hospitalizations, and deaths

	RA			AS			PsA			SLE			SSc		
	Patients	Population referents	IRR	Patients	Population referents	IRR	Patients	Population referents	IRR	Patients	Population referents	IRR	Patients	Population referents	IRR
COVID-19 infection, IR (95% CI)	19.2 (17.9, 20.7)	14.4 (13.9, 15.0)	**1.33 (1.23**, **1.44)**	23.1 (20.3, 26.4)	18.0 (16.9, 19.3)	**1.28 (1.10**, **1.49)**	21.3 (19.0, 23.9)	17.3 (16.4, 18.4)	**1.23 (1.07**, **1.40)**	19.8 (17.2, 22.8)	16.4 (15.3, 17.6)	**1.21 (1.03**, **1.42)**	24.3 (18.4, 32.2)	16.0 (13.7, 18.7)	**1.52 (1.08**, **2.11)**
COVID-19 hospitalization, IR (95% CI)	7.9 (7.1, 8.8)	4.6 (4.3, 4.9)	**1.71 (1.50**, **1.95)**	4.4 (3.2, 5.9)	3.2 (2.8, 3.8)	1.36 (0.94, 1.93)	4.9 (3.8, 6.2)	3.9 (3.5, 4.4)	1.25 (0.93, 1.64)	5.9 (4.6, 7.7)	3.0 (2.6, 3.6)	**2.0 (1.4**, **2.7)**	10.4 (6.8, 16.0)	4.6 (3.4, 6.1)	**2.28 (1.29**, **3.90)**
COVID-19 deaths, IR (95% CI)	2.0 (1.6, 2.5)	1.0 (0.9, 1.2)	**1.91 (1.46**, **2.49)**	0.3 (0.1, 1.0)	0.4 (0.3, 0.7)	0.71 (0.14, 2.39)	0.8 (0.4, 1.4)	0.6 (0.5, 0.8)	1.22 (0.54, 2.47)	0.90 (0.5, 1.74)	0.50 (0.3, 0.8)	1.80 (0.74, 3.98)	2.5 (1.0, 6.0)	0.9 (0.5, 1.7)	2.78 (0.73, 9.23)

IR per 1000 person years with 95% CI for COVID-19 infection, hospitalization and death among RA, AS, PsA, SLE and SSc patients and their matched referents from the general population. Patient-to-population referents IRR with 95% CI have been estimated for each disease group. IRR indicating a statistically significant difference between patients and their matched population referents are denoted in bold. COVID-19: coronavirus disease 2019; IR: incidence rate; IRR: incidence rate ratio.

### Subgroup analyses among SARS-CoV-2-infected subjects

**Fig. 1:**
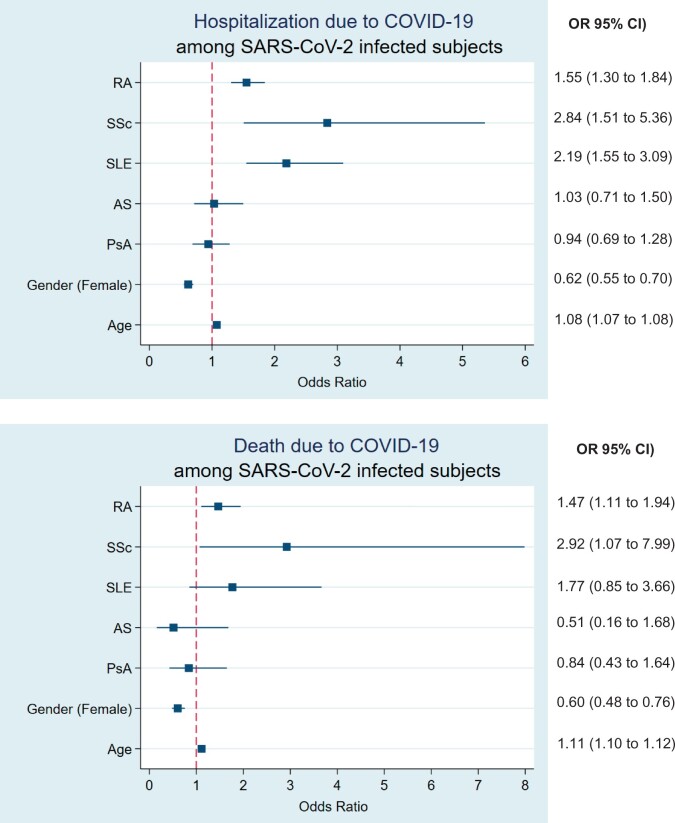
Logistic regression analysis assessing odd ratios for COVID-19-associated hospitalization and death among SARS-CoV-2-infected subjects Odds ratios with 95% CI for hospitalization and death due to COVID-19 focusing only on subjects infected with SARS-CoV-2 between 1 March 2020 and 28 February 2021. Model adjusted for underlying systemic rheumatic disease, age and gender. SARS-CoV-2-infected subjects without underlying systemic rheumatic disease are used as the reference category. COVID-19: coronavirus disease 2019; SARS-CoV-2: severe acute respiratory syndrome coronavirus 2.

To investigate for potential differences between patients with systemic rheumatic disease infected with SARS-CoV-2, we performed a subgroup analysis, using infected subjects without underlying rheumatic disease as the reference group. After adjustment for age and sex, we found that the OR for COVID-19-associated hospitalization was higher in SSc (OR 2.84, 95% CI 1.51, 5.36), followed by SLE (OR 2.19, 95% CI 1.55, 3.09) and RA patients (OR 1.55, 95% CI 1.30, 1.84). Regarding COVID-19-associated death once infected, SSc patients had the worse outcomes in comparison with the general population (OR 2.92, 95% CI 1.07, 7.99), followed by RA patients (OR 1.47, 95% CI 1.11, 1.94). No difference in COVID-19-associated mortality was found between patients with AS, PsA or SLE and the general population (Fig. 1).

### Overall mortality of RA, AS, PsA, SLE and SSc patients during the first pandemic year in comparison with the pre-COVID-19 year

For RA, AS, PsA, SLE and SSc patients in our cohort and their matched referents from the general population we estimated IR of death from all causes per 1000 person-years for the time period under study, as shown in Table 3. The comparison cohort for the year 2019 included 36 589 RA patients [79.9% female, median (Q1–Q3) age 66.7 (56.7–75.2) years], 8527 AS patients [41.5% female, age 50.7 (41.3–59.7) years], 12 025 PsA patients [55.0% female, age 57.4 (47.9–67.3) years], 10 073 SLE patients [89.7% female, age 55.3 (44.3–66.5) years] and 1938 SSc patients [88.4% female, age 61.8 (51.5–70.9) years]. Using all-cause mortality data from the comparison cohort we estimated death rates per 1000 person-years in the pre-COVID-19 era, between 1 January 2019 and 31 December 2019 (Table 3). Interestingly, using 2019 as the reference year, all-cause mortality was higher for RA patients in comparison with the general population during the first year of the pandemic in Greece [IRR (95% CI) for death from all causes: 0.71 (0.67–0.74) in 2020–21 *vs* 0.59 (0.55–0.63) in 2019], whereas it decreased by more than half for SSc patients [IRR (95% CI) for death from all causes: 1.94 (1.74–2.17) in 2020–21 *vs* 4.36 (4.30–4.43) in 2019]. No difference was found for AS, PsA and SLE patients.

**Table 3:** All-cause mortality among patients and population referents during the pandemic (2020–21) and pre-COVID-19 (2019) era

Deaths from all causes	RA			AS			PsA			SLE			SSc		
	IR (95% CI)		IRR (95% CI)	IR (95% CI)		IRR (95% CI)	IR (95% CI)		IRR (95% CI)	IR (95% CI)		IRR (95% CI)	IR (95% CI)		IRR (95% CI)
	Patients	Pop. referents		Patients	Pop. referents		Patients	Pop. referents		Patients	Pop. referents		Patients	Pop. referents	
2020–21	15.1 (13.9, 16.3)	21.2 (20.6, 21.9)	0.71 (0.65, 0.77)	3.8 (2.7, 5.2)	5.8 (5.2, 6.6)	0.65 (0.44, 0.92)	6.1 (4.9, 7.5)	11.5 (10.7, 12.3)	0.53 (0.41, 0.66)	10.0 (8.2, 12.1)	9.6 (8.8, 10.5)	1.04 (0.83, 1.29)	22.3 (16.7, 29.9)	11.5 (9.6, 13.8)	1.94 (1.34, 2.76)
2019	9.0 (8.1, 10.0)	15.3 (14.7, 15.9)	0.59 (0.52, 0.66)	3.4 (2.4, 4.9)	6.0 (5.3, 6.8)	0.57 (0.38, 0.84)	9.2 (7.7, 11.1)	17.9 (16.9, 19.0)	0.51 (0.42, 0.63)	14.1 (12.0, 16.7)	13.8 (12.8, 14.9)	1.02 (0.85, 1.23)	42.3 (34.0, 52.7)	9.7 (7.9, 11.9)	4.36 (3.20, 5.96)

IR per 1000 person years with 95% CI for death from all causes among RA, AS, PsA, SLE and SSc patients and their matched referents from the general population. Data are presented for two different time periods: 1 March 2020 to 28 February 2021, and 1 January 2019 to 31 December 2019. Patient-to-population referents IRR with 95% CI have been estimated for each disease group for the different time periods under study. COVID-19: coronavirus disease 2019; IR: incidence rate; IRR: incidence rate ratio.

## Discussion

Our 12-month study, analysing data derived from merging large nationwide databases covering the entire Greek population, shows that unvaccinated patients with RA, SLE and SSc have a higher probability for hospitalization due to COVID-19, while patients with RA also have a higher COVID-19-associated mortality rate compared with the general population. Given the well-established association of older age with an increased risk of COVID-19-related death, the higher mortality in RA patients, who are older than patients with AS, PsA, SLE and SSc, could be partly explained by the age-associated burden of comorbidities which are known to be more frequent in any systemic rheumatic disease [6–12].

Regarding the risk of SARS-CoV-2 infection, we found that patients with the systemic rheumatic diseases under study had higher probability of contracting COVID-19 compared with the general population, in agreement also with two general population–based cohort studies [14, 15] and two recent meta-analyses [4, 5]. This finding could be explained by higher susceptibility of these individuals to COVID-19 infection although the possibility that these patients were tested more frequently cannot be excluded. Therefore, whether a tendency to contract the infection more easily, together with the increased comorbidity burden, can explain the higher COVID-19-associated death risk found in RA patients compared with the general population needs further study [16]. Along these lines, when we focused only on subjects infected with SARS-CoV-2, adjusting for age and gender, we found that patients with SSc and RA infected with COVID-19 displayed an increased OR both for COVID-19 hospitalization and death compared with the general population.

To investigate whether the pandemic had an effect on all-cause mortality for rheumatic patients, we further analysed the age- and gender-adjusted mortality between 2019 and 2020 across all rheumatic patient groups. This effect of the pandemic can be direct due to COVID-19 infection, but also indirect due to reduced access to care as a result of the stringent public health measures, or any other factor affecting mortality in the population. Our data indicate that in comparison with matched population referents, all-cause mortality for patients with RA increased during the first year of the pandemic compared with 2019, suggesting that COVID-19 affected this population. Recently, a systematic analysis was published, estimating the excess mortality due to the COVID-19 pandemic during the 2-year period 2020–21, in many different countries and territories of the world. According to this analysis, 20 800 deaths due to COVID-19 have been registered in Greece during the first 2 years of the pandemic, leading to a reported COVID-19 mortality rate of 104.1 with an estimated excess mortality rate of 127.1 (95% CI 117.0, 137.2) per 100 000 [17].

On the other hand, all-cause mortality decreased by more than half for patients with SSc. This difference could reflect the extent and efficacy of self-protective measures taken by patients with SSc, a large proportion of them having clinically significant interstitial lung disease [18]. This finding is of particular interest and shows that when vulnerable populations with chronic diseases are compliant with public health measures, all-cause mortality can be reduced during a pandemic. This highlights the importance of non-pharmaceutical interventions among SSc patients and further supports the need for public health-risk communication during a global health crisis. It should be noted that a similarly impressive reduction in all-cause mortality was reported in the same setting, i.e. during the early COVID-19 lockdown in Greece, for patients with idiopathic pulmonary fibrosis, possibly due to the implementation of public health measures against COVID-19, such as social distancing, use of face masks and hand hygiene [19].

To the best of our knowledge only one previous nationwide study assessing mortality risk of unvaccinated patients with chronic inflammatory arthritis *vs* the general population has been published [20]. This was a 6-month study in Sweden, a country where public measures were far less strict, especially during the first pandemic wave, compared with the rest of the world. The authors reported that death rates were increased when adjusting only for age, sex and region of domicile, but this risk was mitigated when additionally adjusting for comorbidities and socioeconomic factors. The adjusted hazard ratio for COVID-19-associated death in all inflammatory joint diseases examined in this study was 1.18 (95% CI 0.97, 1.44), while the adjusted hazard ratio for COVID-19-associated death in RA was 1.27 (1.02, 1.59), compared with matched general population comparator subjects, which is, indeed, significantly higher. The authors concluded that, in absolute terms, risks of serious outcomes from COVID-19 in patients with inflammatory joint disease are strongly affected by age and the presence of comorbidities. Our findings, in accordance with the Swedish nationwide study, also indicate an increased likelihood of death due to COVID-19 among RA patients in comparison with the general population, while no difference could be shown among the other inflammatory arthritis groups examined.

A second nationwide 5-month study from South Korea included 133 609 adult subjects tested for SARS-CoV-2 (3.65% tested positive), of whom 8297 had inflammatory rheumatic diseases. In comparison with the general population, patients with rheumatic disease had a 19% increased likelihood of testing positive for SARS-CoV-2, 26% higher risk of severe COVID-19 outcomes and 69% higher risk of COVID-19-associated death. Patients with rheumatic diseases receiving higher CS doses (≥10 mg prednisolone per day) were particularly vulnerable to developing worse COVID-19-associated outcomes [21]. Notably, our results are in line with this study, as well as with the results of a meta-analysis by Conway *et al.* reporting an OR of 1.74 (95% CI 1.08, 2.80) for COVID-19-related death in systemic rheumatic disease patients in comparison with the general population [5]. It should be here noted that both our study and the meta-analysis of Conway *et al.* could not adjust for comorbidities, disease severity or disease duration, due to the lack of the corresponding data in the data sources used. This important limitation should be taken in account when interpreting these findings, given the high frequency of comorbidities among patients with systemic rheumatic disease [6–12] and the strong influence that comorbidities and disease activity exert on COVID-19-associated outcomes in this patient population [22, 23].

As regards hospitalization rates, there are four nationwide studies, one from Sweden [20], two from Denmark [15, 24] and one from Iceland [25], in line with our findings, while two meta-analyses [4, 5] report different results on this matter. This could be interpreted in light of several differences between countries, such as local guidelines, intensity of pandemic wave, saturation level of healthcare system, access to healthcare facilities and other confounders [26]. Increased hospitalization rates could be possibly also due, at least in part, to lower threshold for admission of patients suffering from a systemic rheumatic disease. Besides, in concert with our results various factors have been previously identified to associate with COVID-19-related hospitalization, including male gender, higher age and specific diseases like RA, vasculitis and CTD [23, 24, 27–30]. Importantly, vaccination [15] and treatment with biologic agents has been negatively associated with hospital admission in other studies [4, 28, 31].

In general, findings about COVID-19-related outcomes in systemic rheumatic disease patients should be interpreted with caution [32]. Inconsistencies between various studies may pertain to ethnic or racial differences [33], to the heterogeneity of systemic rheumatic disease and their treatment, differences in the intensity of pandemic waves and the capacity of healthcare system, but also to the different study designs or timing of data acquisition with regard to the implementation of patient vaccination policies [15]. That said, we should note that our study was performed before vaccination was available among systemic rheumatic disease patients in Greece.

A recently published study also raised some concerns about a high rate of bias (e.g. participation and ascertainment bias) occurring in the studies being published about this topic [34]. This was also noted in a recent systematic literature review performed to inform respective EULAR recommendations [3]. Among the strengths of our study, one can include the following: firstly, it is a nationwide study examining the whole population of our country (∼11 million people) during the whole first year of the pandemic. To further strengthen our methodology, matching with the general population was adjusted for area of residence, limiting biases concerning access to healthcare facilities and regional differences in COVID-19 incidence rate. Despite the fact that population coverage of the IDIKA database is very high, there is always a risk that patients with a lower socioeconomic status, usually being followed up in public hospitals, are overrepresented in the database compared with patients from the private sector, who would expectedly be able to afford to buy their medications. To eliminate selection bias, we limited our requirements for inclusion in the cohort to one filled prescription with any of the above-mentioned medications of interest between 2015 and 2019. Therefore, only a very small minority of patients consistently buying their rheumatology drugs over the counter for five consecutive years would not have been captured. Also, in contrast to other studies, we have examined simultaneously five major systemic rheumatic diseases in the same population, which also allowed us to make comparison between diseases.

One major weakness of our study, as mentioned above, is the inability to adjust our findings for disease duration, disease activity and the presence of comorbidities. However, in the sense that comorbidities now tend to be seen as an inherent component of systemic rheumatic disease, we believe that it is hard to decipher whether the higher COVID-19 associated death rate observed in certain of these patient’ subgroups should be attributed to the systemic rheumatic disease *per se* or to the concomitant presence of other diseases. It is also worth mentioning that no adjustment was made in our analysis for use of rheumatic disease specific treatments, such as corticosteroids, conventional synthetic, targeted synthetic or biologic DMARDs. Some previous studies have, indeed, reported an association of certain treatments, namely CS at a dose of ≥10 mg prednisolone/day [28], or rituximab [35], with a worse COVID-19 outcome, while other medications were found to have a beneficial effect, with tocilizumab [36] or baricitinib [37], for example, now being used to treat severe COVID-19 infections. Although data on medication usage for our cohort could be retrieved from the database, other potential confounders such as disease duration, disease severity, comorbidities, treatment adherence or timing of treatment with regard to the occurrence of SARS-CoV-2 infection could not be addressed, which would have undermined the robustness of our results. Therefore, we deemed it best not to include medication usage in our analysis.

### Conclusions

COVID-19 outcomes clearly differ between patients with RA, AS, PsA, SLE and SSc. The probability of death from COVID-19 was found higher in RA patients *vs* matched referents from the general population. Furthermore, once infected with SARS-CoV-2, both RA and SSc patients had worse COVID-19-associated outcomes compared with the general population, implying that priority vaccination policies and access to targeted therapeutic approaches are important, especially for older patients and specific patient subgroups. The increased death rate is probably attributable to the presence of comorbidities, which are inherent to the chronic inflammatory process characterizing systemic rheumatic diseases. Further studies could help in effective risk stratification for systemic rheumatic disease patients and therefore result in better outcomes.

## Supplementary Material

keac422_Supplementary_Data

## Data Availability

The data that support the findings of this study are available from the Data Protection Officer of the Greek Ministry of Health but restrictions apply to the availability of these data, which were used under license for the current study, and so are not publicly available. Data are however available from the authors upon reasonable request and with permission of the Data Protection Officer of the Greek Ministry of Health.
