# Supplementary material for: Different COVID-19 outcomes among systemic rheumatic diseases: a nation-wide cohort study
Source: Rheumatology (Oxford). 2022 Aug 3;62(3):1047–56. doi: 10.1093/rheumatology/keac422 (PMC9384656; doi:10.1093/rheumatology/keac422)
Supplement: keac422_Supplementary_Data [file keac422_supplementary_data.docx]

**Supplement**

**Table S1.** Predefined ICD-10 codes used to identify patients in each group of the systemic rheumatic diseases under study.

| Rheumatoid Arthritis | M05, M05.0, M05.1, M05.2, M05.3, M05.8, M05.9, M06, M06.0, Μ06.2, Μ06.3, M06.4, M06.8, M06.9 |
| --- | --- |
| Ankylosing Spondylitis | M45, Μ46, Μ46.0, Μ46.1, Μ46.8, Μ46.9, Μ07.2 |
| Psoriatic Arthritis | M07, Μ07.0, Μ07.1, Μ07.3, L40.5 |
| Systemic Lupus Erythematosus | M32, M32.0, M32.1, M32.8, M32.9 |
| Systemic Sclerosis | M34, M34.0, M34.1, M34.2, M34.8, M34.9 |

**Table S2.** ATC5 codes of medications used to identify patients in each group of the systemic rheumatic diseases under study

| **Medication Class** | **ATC5 code** | **Medication** |
| --- | --- | --- |
| conventional synthetic (cs)DMARDs | A07EC01 | sulfasalazine |
|  | L01BA01 | methotrexate sc |
|  | L04AX03 | methotrexate per os |
|  | L04AA13 | leflunomide |
|  | P01BA02 | hydroxychloroquine |
|  | M01CC01 | D-penicillamine |
| Immunosuppressants | L04AD01 | ciclosporine |
|  | L04AX01 | azathioprine |
|  | L01AA01 | cyclophosphamide |
|  | L04AA06 | mycophenolic acid |
| corticosteroids | H02AB01 | betamethasone |
|  | H02AB02 | dexamethasone |
|  | H02AB03 | fluocortolone |
|  | H02AB04 | methylprednisolone |
|  | H02AB05 | paramethasone |
|  | H02AB06 | prednisolone |
|  | H02AB07 | prednisone |
|  | H02AB08 | triamcinolone |
|  | H02AB09 | hydrocortisone |
|  | H02AB10 | cortisone |
|  | H02AB11 | prednylidene |
|  | H02AB12 | rimexolone |
|  | H02AB13 | deflazacort |
|  | H02AB14 | cloprednol |
|  | H02AB15 | meprednisone |
|  | H02AB17 | cortivazol |
| biologic (b)DMARDs | L04AA24 | abatacept |
|  | L04AC07 | tocilizumab |
|  | L04AC03 | anakinra |
|  | L04AC05 | ustekinumab |
|  | L04AC10 | secukinumab |
|  | L01XC02 | rituximab |
|  | L04AB04 | adalimumab |
|  | L04AB05 | certolizumab pegol |
|  | L04AB01 | etarnecept |
|  | L04AB06 | golimumab |
|  | L04AB02 | infliximab |
|  | L04AA26 | belimumab |
| targeted synthetic (ts)DMARDs | L04AA29 | tofacitinib |
|  | L04AA44 | upadacitinib, |
|  | L04AA37 | baricitinib, |
|  | L04AA32 | apremilast |
| advanced vasodilatory therapy | C02KX01 | bosentan |
|  | C02KX02 | ambrisentan |
|  | C02KX03 | sitaxentan |
|  | C02KX04 | macitentan |
|  | C02KX05 | riociguat |
|  | C02KX52 | ambrisentan and tadalafil |
|  | G04BE08 | tadalafil |
|  | G04BE03 | sildenafil |
|  | G04BE09 | vardenafil |
|  | G04BE10 | avanafil |
|  | G04BE11 | udenafil |
|  | B01AC09 | epoprostenol |
|  | B01AC11 | iloprost |
|  | B01AC19 | beraprost |
|  | B01AC21 | treprostinil |
|  | B01AC27 | selexipag |
| antifibrotic therapy | L01XE31 | nintedanib |
|  | L04AX05 | pirfenidone |
